# Biological and Medicinal Properties of *Chrysanthemum boreale* Makino and Its Bioactive Products

**DOI:** 10.3390/ijms26135956

**Published:** 2025-06-20

**Authors:** Christian Bailly

**Affiliations:** 1 UMR9020-U1277-CANTHER-Cancer Heterogeneity Plasticity and Resistance to Therapies, CHU Lille, CNRS, Inserm, OncoLille Institut, University of Lille, 59000 Lille, France; christian.bailly@univ-lille.fr; 2 Institute of Pharmaceutical Chemistry Albert Lespagnol (ICPAL), Faculty of Pharmacy, University of Lille, 59006 Lille, France; 3 OncoWitan, Scientific Consulting Office, 59290 Lille, France

**Keywords:** *Chrysanthemum*, *Chrysanthemum boreale*, cumambrin, handelin, anti-inflammatory, anti-infective, anticancer

## Abstract

*Chrysanthemum* species represent an economically important group of flowering plants. Many species also present a medicinal interest, notably for the treatment of inflammatory pathologies. This is the case for *Chrysanthemum boreale* Makino, endemic to Japan and widespread in Eastern Asia. This perennial plant has long been used in folk medicine to treat inflammatory diseases and bacterial infections. An extensive review of the scientific literature pertaining to *C. boreale* has been performed to analyze the origin of the plant, its genetic traits, the traditional usages, and the properties of aqueous or organic plant extracts and essential oils derived from this species. Aqueous extracts and the associated flavonoids, such as acacetin and glycoside derivatives, display potent antioxidant activities. These aqueous extracts and floral waters are used mainly as cytoprotective agents. Organic extracts, in particular those made from methanol or ethanol, essentially display antioxidant and anti-inflammatory properties useful to protect organs from oxidative damage. They can be used for neuroprotection. Essential oils from *C. boreale* have been used as cytoprotective or antibacterial agents. The main bioactive natural products isolated from the plant include flavonoids such as acacetin and related glycosides (notably linarin), and diverse sesquiterpene lactones (SLs). Among monomeric SLs, cumambrins and borenolide are the main products of interest, with cumambrin A targeting covalently the transcription factor NF-κB to regulate proinflammatory gene expression to limit osteoclastic bone resorption. The dimeric SL handelin, which is characteristic of *C. boreale*, exhibits a prominent anti-inflammatory action, with a capacity to target key proteins like kinase TAK1 and chaperone Hsp70. A few other natural products isolated from the plant (tulipinolide, polyacetylenic derivatives) are discussed. Altogether, the review explores all medicinal usages of the plant and the associated phytochemical panorama, with the objective of promoting further botanical and chemical studies of this ancestral medicinal species.

## 1. Introduction

Plants from the genus *Chrysanthemum* (family: *Asteraceae* (Compositae)) represent an economically important group of floricultural crops worldwide. They are the second most important and popular group of ornamental plants after roses [1]. In 2022, *Chrysanthemums* represented about 10% of the global cut flower trade market, with a hybrid *Chrysanthemum* seeds market estimated at USD 1437 million and an annual growth rate of 7.7% from 2023 to 2032 [2,3]. *Chrysanthemum* plants are considered an emblem of longevity and good fortune in Japan [4]. New varieties are continuously developed to promote their ornamental and economic values, such as the development of cold-tolerant subspecies, blue-colored cultivars, or plant hybrids with new shapes, sizes, floral scents, or flowering times [5,6]. Transgenic molecular breeding methods are used to produce new species resistant to insects, viruses, and other pathogens [7,8,9]. In parallel, conventional propagation techniques and alternative in vitro propagation strategies for *Chrysanthemum* continue to be developed to generate new varieties [10].

*Chrysanthemum* species represent an important group of medicinal plants. They can be used as a source of nutrients, vitamins, minerals, and secondary metabolites including phenolic compounds, flavonoids, and alkaloids [11,12]. For example, the species *Chrysanthemum morifolium* Ramat. is used to prepare flower teas (hangbaiju and hangju in Chinese; hangeul and hanja in Korean) with antioxidant and anti-inflammatory properties [13,14]. The plant contains a variety of natural products beneficial to health, notably polysaccharides to treat constipation, colitis, and other digestive pathologies [15,16]. Similarly, the species *C. indicum* L. (synonym: *C. nankingense*) is used in traditional medicine for its detoxifying properties to treat swelling, pain, scrofula, and other ailments [17,18]. Beyond these two extensively studied species originating from Asia, there are other less-known *Chrysanthemum* species of interest such as the short-day flowering species *C. vestitum* [19] and the species *C. arcticum* native to North America, to cite only two examples [20]. An analysis of the phylogenetic relationship between >30 *Chrysanthemum* species has identified two major subgroups based on morphological characteristics [7,21]. However, there are a myriad of varieties in this family and newly emerging categories, which require sophisticated visualization methods for their precise identification and classification [13,22,23].

**Figure 1:**
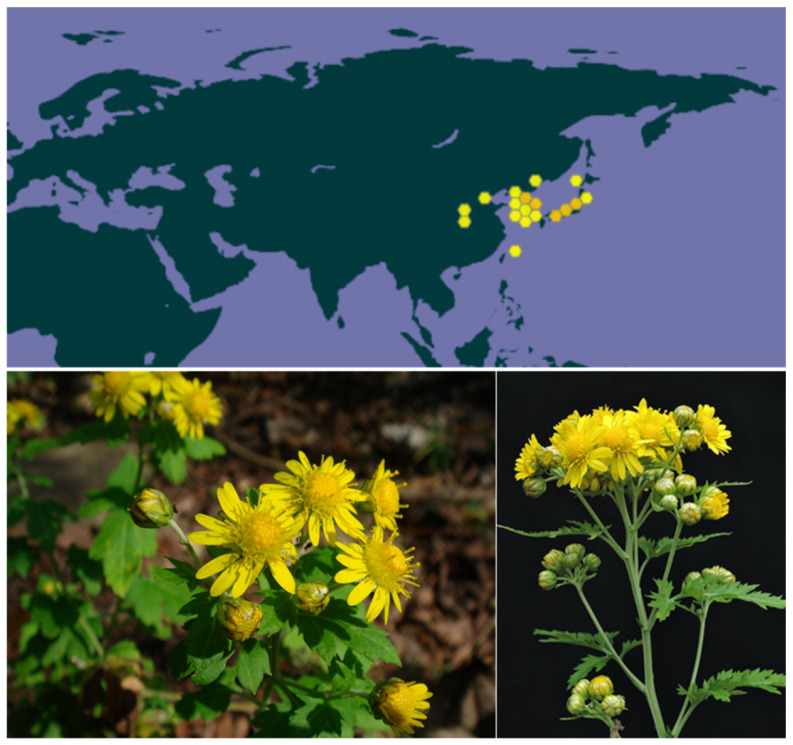
Distribution and illustrations of *Chrysanthemum boreale* Makino. The distribution map was obtained from the Global Biodiversity Information Facility (GBIF) (https://www.gbif.org/species/10667426) (accessed on 2 June 2025). Yellow and orange dots indicate the presence of the plant. The two photos of the plants are from Prof. Summer’s Web Garden (http://flowers.la.coocan.jp/Asteraceae/Chrysanthemum%20seticuspe%20boreale.htm (accessed on 2 June 2025).

One particular *Chrysanthemum* species has caught the attention of scientists: *Chrysanthemum boreale* Makino (hereafter *C. boreale*) which is a cold-resistant perennial species native to Eastern Asia—essentially Korea, China, and Japan—with a significant medicinal and phytochemical interest [24] (Figure 1). Potent bioactive compounds have been isolated from this plant, in particular guaianolide-type sesquiterpene lactones with anti-inflammatory properties. The present review provides an updated overview of the plant, its medicinal properties, and the natural products that are the origin of the reported pharmacological effects. The phytochemical survey is focused on *C. boreale*, to highlight the most interesting natural products and their pharmacological properties. Particular attention is given to monomeric and dimeric guaianolides isolated from the plant, their properties, and their mechanism of action.

## 2. The Plant *Chrysanthemum boreale* Makino

### 2.1. Distribution and Morphology

The species *C. boreale* Makino was originally described in the Botanical Magazine (Shokubutsu-gaku zasshi, Tokyo) in the early twentieth century [24]. The original name was *Chrysanthemum indicum var. boreale* Makino, now referred to as *Chrysanthemum boreale* (Makino) Makino (accepted name) and occasionally under the synonym name *Chrysanthemum seticuspe* (Maxim.) Hand.-Mazz., or *Chrysanthemum lavandulifolium* (Fisch. ex Trautv.) Makino (WFO). The plant is endemic to Japan and well distributed in several provinces (South of Tohoku, Kanto, Kinki, North of Kyushu), being known under the common Japanese names kikutani-giku or awa-kogane-giku for bubbling golden daisy [25,26].

It is a perennial plant, relatively tall, up to 100–150 cm, with small leaves (5–7 cm length × 4–6 cm width). It produces nice yellow flowers in October–November. Wild plants of heights up to 200–245 cm have been reported as well [27]. Wild *C. boreale* is also well distributed in the mountainous regions of the Republic of Korea where it is widely used as an ornamental plant [28]. It can be found in Gangwon-do, Gyeonggi-do, Gyeongsangbuk-do, Gyeongsangnam-do, and Jeollabuk-do provinces in Korea [29,30]. The dried plant is used to prepare a green tea-like beverage in Korea known as kukwha tea, which contains volatile sesquiterpenes such as santalol and farnesene, and terpenes such as thymol [31].

To satisfy the increasing demand for *C. boreale*, processes have been developed to increase plant growth, notably via nitrogen fertilization [32,33]. The flower biomass yield can reach ~2450 kg/ha when using swine manure, poultry manure, fly ash, or other fertilizers [34,35,36]. The planting time and fertilization strategy are key parameters for the production of many *Chrysanthemum* species [37]. Notably, fertilization with calcium carbonate has been evidenced as a key parameter to improve the yield and quality of the flower in *C. boreale*. A field treatment with CaCO_3_ (up to 1.5 ton/ha) increased significantly the total plant yield and the content in sesquiterpenes (+30.4%) and monoterpenoids (+9.5%) in dry weight of the flowers [38,39].

### 2.2. Genomic Content

The complete sequencing of both the chloroplast and mitochondrial genomes of *C. boreale* has revealed the presence of 131 and 58 genes, including 87 and 35 protein-coding genes, respectively. The phylogenetic analysis indicated that the chloroplast genome of *C. boreale* is very close to that of species *C. indicum* and *C. morifolium*, whereas the mitogenome of *C. boreale* rather corresponds to that of other *Asteraceae* such as *Helianthus annuus* [40,41]. Nevertheless, a comparative transcriptomic analysis has suggested that *C. boreale* diverged from *C. morifolium* about 1.7 million years ago [42,43].

Another study of the chloroplast genome of three different strains of *C. boreale* identified 80 unique protein-coding genes and about 300 single nucleotide polymorphisms (SNPs), for a total size of 151,000 base pairs (three strains analyzed: 151,012, 151,098, and 151,010 bp exactly) [43]. It is a diploid species (2n = 18) that can be used to generate hybrid plants by artificial cross-pollination, for example, with the hexaploidy species *C. vestitum* (2n = 54) [44,45]. Alternatively, polyploidy can be induced upon treatment of in vitro shoots with a chemical mutagen such as colchicine to produce tetraploid plants and chimera with distinct characteristics. The tetraploid plants exhibited thicker and larger leaves than the diploid plants [46]. The genome of *C. boreale* contains also a large proportion of repetitive DNA sequences, notably long terminal repeat retrotransposons, dispersed in the entire plant chromosomes and contributing to the genomic regulation [47,48]. The complete and contiguous genome of *C. boreale* has been assembled recently, representing 3.1 Gb of its sequence into nine pseudochromosomes [49]. *C. boreale* is thought to be an important genetic resource for developing new disease-resistant cultivars [50,51].

## 3. Plant Extracts and Essential Oils

**Figure 2:**
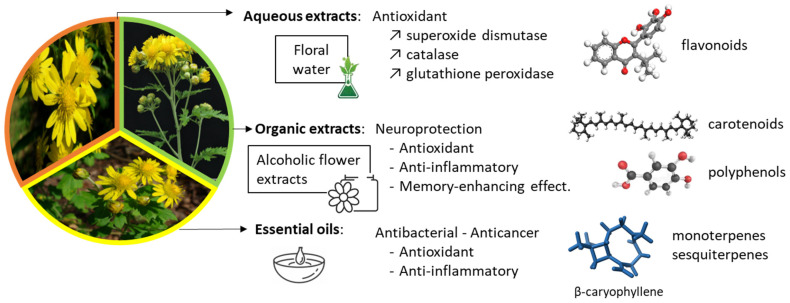
Bioactivities evidenced with various extracts of *Chrysanthemum boreale* Makino. See Table 1, Table 2 and Table 3 for more specific information.

Various types of extracts have been prepared from *C. boreale* to evaluate their pharmacological properties. Schematically, the phytochemical preparations can be divided into three groups, discussed in turn hereafter (Figure 2).

### 3.1. Aqueous Extracts

The whole plant or specific plant parts have been used to prepare aqueous extracts (AEs). For example, AEs have been obtained simply by incubating dry shoots (100 g) in distilled water (1 L) for 24 h prior to filtration. This AE showed a dose-dependent capacity to inhibit shoot and root elongation of various plants. It reduced plant germination, root hair development, and growth, providing thus evidence for an allelopathic action [52]. In another study, the authors prepared an AE from air-dried powdered flowers with water under reflux and this extract showed hepatoprotective effects [53]. AEs of *C. boreale* have shown antioxidant effects, notably by increasing the activity of antioxidant enzymes like superoxide dismutase (SOD), catalase, and glutathione peroxidase [54]. This type of AE generally contains multiple bioactive polar substances. Flavonoids have been characterized from AEs, as well as other active molecules such as guanosine identified in crude water extracts of the flowers of *C. boreale* and possibly responsible for the inhibitory activity of the angiotensin-converting enzyme (ACE) [55].

Other types of AE extracts have been obtained via a steam distillation process, which consisted of a passage of vapor from a boiler into a chamber holding the plant, followed by condensation of the solute-containing steam with cold water. The process affords an aqueous extract (separated from the hydrophobic essential oil) containing volatile compounds such as cyclohexanone derivatives, thymol, eugenol, carveol, and other compounds with antioxidant and anti-inflammatory effects. The floral water thus obtained revealed a capacity to inhibit migration and proliferation of aortic smooth muscle cells, suggesting its beneficial use to treat vascular disorders [56]. It is interesting to note that the antioxidant effect of an AE from *C. boreale* was found to be significantly superior to the same AE prepared from the related species *C. zawadskii* and *C. indicum* (4.37–4.50, 3.44–3.55 and 2.51–2.63 mg AA eq/g (L-ascorbic acid equivalent), respectively) [57]. Water extracts from *C. boreale* display both antioxidant and anti-inflammatory effects, but the potency of those extracts is generally inferior to that observed with organic extracts prepared with methanol, ethyl acetate, or chloroform [58]. Diverse effects have been reported with *C. boreale* AEs, including hepatoprotection, immunomodulation, antioxidant, and vascular modeling (Table 1).

**Table 1:** Pharmacological effects reported with aqueous extracts (AEs) of *C. boreale*.

Activity Types	Main Observations	References
Hepatoprotection	Reduction of CCl_4_-induced hepatic damages in mice with an AE orally given. The AE decreased the levels of serum liver enzymes (aspartate aminotransferase, alanine aminotransferase, lactate dehydrogenase, and alkaline phosphatase).	[53]
Antioxidant activity	Oral administration of the AE increased activity of antioxidant enzymes (SOD, catalase, and glutathione peroxidase), and the concentration of dopamine in brain of Parkinson-type mice.	[54]
Vascular modeling	A floral water from *C. boreale* inhibited migration and proliferation of aortic smooth muscle cells. The extract modulated the MAPK pathway through inhibition of PDGFR-β.	[56]
Anti-atopic dermatitis	Reduction in skin symptom severity and inflammation in a mouse model of atopic dermatitis. The methanolic extract decreased expression of TNF-α, IL-4, and the level of serum IgE in mice orally treated with the plant extract.	[59]
Anti-diabetic	A water extract of *C. boreale* flowers showed a marked inhibitory activity of angiotensin-converting enzyme (ACE). Guanosine was identified as the main inhibitor.	[55]
Skin regeneration	Effect of a hydrosoluble extract of *C. boreale* on skin regeneration. The hydrosol promoted proliferation and migration of human HaCat skin keratinocytes.	[60]
Plant growth Inhibition	*C. boreale* AE reduced plant germination, root hair development, and growth of various plants (allelopathic effect).	[52]

### 3.2. Organic Extracts

Different types of pharmacological activities have been reported with organic extracts (OEs) of *C. boreale*, as indicated in Table 2. In most cases, these extracts were prepared using ethanol, occasionally with chloroform or another apolar solvent. The whole plant or specific parts of the plant were used, depending on the objective of the study. For example, the extraction of phenolic compounds from petals of *C. boreale* was optimized using 98% ethanol (115 mL/g) for 16 h, whereas carotenoids were efficiently extracted with 75% ethanol (143 mL/g) for 19 h [61]. In another study, the authors utilized an ethanolic flower extract to evidence the memory-enhancing capacity of the preparation in scopolamine-treated mice having cognitive and memory impairment, to model Alzheimer’s disease. In this case, the extract (diet-administered) alleviated memory deficits by modulating neurotransmitters and expression of proteins ERK1/2 related to synaptic function in a scopolamine-treated animal model [62]. Other studies used methanolic extracts of the whole dried plant to investigate the antioxidant capacity and neuroprotective effects of the extract. A marked reduction in oxidative damage was observed in an in vitro model of stress-induced neurotoxicity. The extract reduced the H_2_O_2_-induced death of SH-SY5Y cells by preventing the activation of caspase-3 and the MAPK/CREB pathway [63]. In the same vein, a methanolic extract of *C. boreale* showed potent anti-inflammatory activity in RAW264.7 macrophages with a capacity to reduce expression and activity of heme oxygenase-1 (HO-1) in a dose-dependent manner, together with inhibition of nitric oxide (NO) production and expression of inducible nitric oxide synthase (iNOS) protein [64].

The use of chloroform as an extraction solvent led to extracts with a higher content in hydrophobic substances, notably sesquiterpenoid lactones endowed with marked antibacterial effects, against pathogenic bacteria like *Vibrio parahaemolyticus* (Gram-negative) and *Bacillus subtilis* (Gram-positive) [65]. The whole plant extract showed a superior antibacterial activity than the flower extract [66]. The CHCl_3_ fraction from *C. boreale* displayed marked cytotoxicity against L1210, K562, and A549 tumor cells, with ED_50_ values of 3.98, 4.28, and 3.84 µg/mL, respectively (compared to 0.02, 0.18, and 1.54 µg/mL with the reference drug 5-fluorouracil) [67].

**Table 2:** Pharmacological effects reported with organic extracts (OE) of *C. boreale*.

Activity Types	Main Observations	References
Neuronal protection	Reduction in neuronal damages in vitro with a methanolic extract of *C. boreale.*	[63]
Antioxidant activity	Potent antioxidant activity of a methanolic extract of flowers from *C. boreale*.	[68]
Protection against retinal damages	An ethyl acetate fraction prepared from *C. boreale* flowers showed potent antioxidant activity in retinal pigment epithelium cells.	[69]
Anti-inflammatory activity	Marked inhibition of NO production and iNOS expression with a methanolic extract of *C. boreale*.	[64]
Antibacterial Effects	A chloroform extract revealed antibacterial effects against selected bacterial strains. Sesquiterpenoid lactones were identified from the extract.	[65]
Cytotoxic activities	Antiproliferative activity of a chloroform extract of *C. boreale* against K562 human myeloid leukemia cells and isolation of an active substance.	[67]

### 3.3. Essential Oils

**Figure 3:**
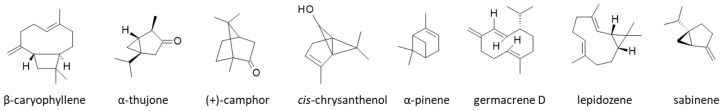
Examples of volatile molecules found in *C. boreale* essential oil (EO).

Essential oils (EOs) are usually obtained by steam distillation of the aerial parts of *C. boreale* using a Clevenger-type apparatus, for about 3 h [70]. A hydrodistillation process is commonly used to prepare EOs, which are then analyzed by gas chromatography–mass spectrometry (GC/MS) to identify the volatile constituents [71]. This type of analysis performed with an EO from *C. boreale* revealed the presence of 87 constituents, including many non-oxygenated or oxygenated monoterpenes and sesquiterpenes, a few aldehydes, and diverse other small molecules (thymol, carvacrol). The EO showed antibacterial activities against some Gram-positive bacteria (including *Staphylococcus aureus* and *Streptococcus pyogenes*) and a few Gram-negative bacteria including *Escherichia coli* [69]. One of the major constituents was the sesquiterpene β-caryophyllene, which is a common anti-inflammatory and antioxidant compound found in many essential oils. This natural product is approved by the FDA (Food and Drug Administration) as a food additive with a GRAS (Generally Recognized as Safe) status [72]. β-caryophyllene (Figure 3) is present in *C. boreale* EOs and has been shown to exert multiple pharmacological effects, including an antiproliferative action against cultured human lung cancer cells [73]. Its chemopreventive action has been underlined in multiple studies [74].

*C. boreale* EO is able to trigger apoptosis of human oral epidermoid carcinoma KB cells, with the typical activation of PARP proteins, induction of DNA fragmentation, and formation of apoptotic bodies in vitro [75]. It is interesting to note that antiproliferative activity has been observed with cancer cells, whereas stimulation of proliferation was observed when using human HaCaT keratinocytes. In this latter case, the *C. boreale* EO induced phosphorylation of Akt and ERK1/2 and promoted wound healing in human skin [76]. EOs may help to promote skin re-epithelization and restore the functions of damaged skin. For this reason, they could be interesting ingredients for cosmetic products. A cosmetic cream containing 0.1% *C. boreale* EO has been shown to improve skin wrinkles (reduction in the roughness index) [77].

The compositions of EOs made from *C. boreale* and the closely related species *C. indicum* were found to be slightly different. A GC/GC-MS analysis revealed the presence of 94 components in the former EO versus 80 in the latter one. Camphor, *cis*-chrysanthenol, and α-thujone (Figure 3) were the main three components in the *C. boreale* EO (15.4%, 14.1%, and 13.3%, respectively) whereas the *C. indicum* EO contained principally germacrene D, camphor, and α-thujone (16.5%, 10.0%, and 6.4%, respectively) [78]. A similar composition has been reported in a recent study of the composition of a *C. boreale* EO, with camphor as the main constituent (20.9%). In this case, the EO was found to potently inhibit biofilm formation and bacterial adherence of the cariogenic bacterial species *Streptococcus mutans* [79]. A slightly different composition was reported in another study, with a prevalence of α-pinene (which exhibits marked anti-cariogenic effects [80]), α-thujone, chrysanthenone, and other compounds [81,82]. In fact, the composition of the EO and hence its properties can vary according to the harvesting stage of *C. boreale*. For example, α-thujone is well present before flowering (5.74%) but little present when flowers begin to open (0.58%) or when they are fully open (0.71%) [80]. During the extraction/purification process, the yield of EO is much higher in the pre-flowering stage compared to the vegetative state. The content in camphor, sabinene, and phellandrene increases with increasing flowering degree, whereas the content in β-caryophyllene, germacrene D, and lepidozene decreases with increasing flowering degree. Moreover, the processing scheme, notably the drying temperature of *C. boreale* flowers, can affect the composition of the EO and the extent of volatile compounds contained in the dried flowers [83]. For different reasons, the aroma compounds and hence the sensory attributes can vary from one *Chrysanthemum* EO to another [84,85]. As a consequence, the biological properties of the EO, notably the skin-whitening activity, can vary depending on the plant harvesting stage. The EO made from the fully-flowering plant showed a superior capacity to induce phosphorylation of p38 MAPK (p38 mitogen-activated protein kinase) in B16BL6 melanoma cells compared to the EO derived from the vegetative plant, thus affecting differently the melanogenic pathway [56,86].

An EO made from *C. boreale* flowers was shown to reduce the extent of skin lesions in a murine model of 2,4-dinitrochlorobenzene (DNCB)-induced dermatitis. The product inhibited TNF-α-induced downregulation of the skin barrier-related proteins filaggrin and loricrin, probably via attenuation of the expression of the SNARE (soluble N-ethylmaleimide-sensitive factor activating protein receptor) proteins (e.g., Vamp8, syntaxin-4) and interference with SNARE protein-associated mast cell degranulation [87]. The effect can be associated with the presence of 1-iodohexadecane, which is a component of *C. boreale* EO and has been shown to inhibit the expression of Vamp8 (vesicle-associated membrane proteins 8) protein and to enhance the expression of filaggrin and loricrin in HaCaT cells. When applied topically to DNCB-lesioned dorsal skin, 1-iodohexadecane (50–100 µg/mL) was found to reduce epidermal thickness and mast cell infiltration, and to increase filaggrin and loricrin expressions [88]. *C. boreale* EO can be used for multiple purposes (Table 3). Drug treatments can also modulate the plant growth and EO content in *C. boreale*. For example, a soil treatment and foliar application of the triazole-type growth regulator uniconazole (a gibberellin synthesis inhibitor) has been shown to increase the production of *C. boreale* EO and to affect its chemical composition [35,89].

**Table 3:** Pharmacological effects reported with essential oils (EOs) from *C. boreale*.

Activity Types	Main Observations	References
Antibacterial activity	Activity of an EO from *C. boreale* against selected bacteria., including *Staphylococcus aureus* and *Streptococcus pyogenes*(Gram-(+)) and *Escherichia coli* (Gram-(−)).	[70]
Anti-biofilm formation	Inhibition of biofilm formation and bacterial adherence by the EO.	[79]
Skin regeneration	Stimulation of keratinocyte proliferation and promotion of wound closure with a *C. boreale* EO.	[76]
Anti-atopic dermatitis	Anti-inflammatory effects, with inhibition of IL-6 production in HaCaT cells.	[90]
Anticancer effects	Inhibition of proliferation and induction of apoptosis of KB cells in vitro with *C. boreale* EO.	[75]
Anti-obesity	Inhibition of lipid accumulation in 3T3-L1 cells by a *C. boreale* EO, via suppression of activation of the adipogenic transcription factors PPAR-γ, C/EBPα, and SREBP-1. Antiadipogenic and lipolysis effects.	[91]
Prevention of muscle atrophy	*C. boreale* EO reduces skeletal muscle atrophy and the monoterpene sabinene is primarily responsible for the effect via regulation of the MAPK/MuRF-1 pathway.	[92]

## 4. Bioactive Substances Isolated from *Chrysanthemum boreale*

### 4.1. Flavonoids

**Figure 4:**
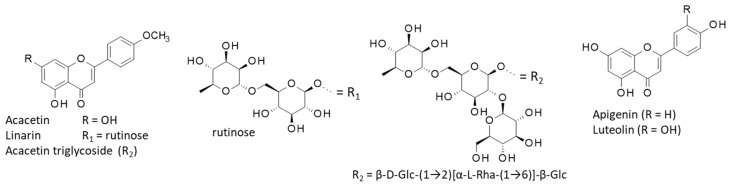
Structures of selected flavonoids found in *C. boreale*: acacetin (5,7-dihydroxy-4′-methoxyflavone), linarin (acacetin-7-*O*-β-D-rutinoside; the rutinosyl (*6-O-α-L-*rhamnosyl-(1→6)-β-D-glucosyl) residue is attached at the 7-OH position) and the triglycoside acacetin 7-*O*-β-D-glucopyranosyl-(1→2)[α-L-rhamnopyranosyl-(1→6)]-β-D-glucopyranoside. Apigenin (4′,5,7,-trihydroxyflavone), and luteolin (3′,4′,5,7-tetrahydroxyflavone).

Diverse natural products have been isolated from *C. boreale* including terpenes, sesquiterpenes, and flavonoids, such as the flavones acacetin (aglycone) and glycoside derivatives such as the diglycoside linarin and a rare triglycoside derivative (Figure 4). Linarin is one of the most abundant flavones, notably in the plant’s leaves and flowers (11.93 and 8.50 mg/g, respectively) [93]. It can be found in *Asteraceae* and in *Lamiaceae* (e.g., *Mentha* [94]), and Scrophulariaceae (e.g., *Linaria* [95]). Linarin and the aglycone acacetin exhibit sedative and anticonvulsant activities, thus possibly explaining the efficiency of the plant in treating stress and anxiety. Linarin exerts sedative effects through inhibition of acetylcholinesterase (AChE) and this effect likely contributes to the anti-cholinesterase activity of the plant extract [96,97,98]. Linarin also presents anti-inflammatory and antioxidant activities of interest to treat osteoporosis [99]. The acacetin triglycoside derivative is an inhibitor of AChE but is much less active than the parent aglycone [94]. The same acacetin trioside has been found in a few totally distinct plants (e.g., *Robinia pseudoacacia*, *Artemisia capillaris*) [100,101].

These three major flavonoids found in *C. boreale* likely contribute to the cytoprotective activity, notably against retinal damage (Kim et al., 2022) [69]. A few other flavonoids have been found in flowers of *C. boreale*, notably apigenin, apigenin-7-*O*-glucuronide, and luteolin [102,103]. This latter compound proved to be a potent inhibitor of aldose reductase (AR) (IC_50_ = 0.5 µM), 60 times more potent than apigenin, whereas linarin is inactive against the same enzyme [104]. AR is an enzyme largely implicated in the etiology of diabetes mellitus (type 2) and related complications (e.g., cataract, macular degeneration). Apigenin is thus possibly responsible for the protection against retinal damages observed with *C. boreale* extracts [69]. Both luteolin-7-*O*-rutinoside and acacetin-7-*O*-rutinoside have been detected in *C. boreale*. They possibly contribute to inhibition of prostaglandin E2 (PGE2) production [105]. There may be a possibility of exploiting an acid extract of *C. boreale* for the treatment or prevention of eye diseases or eye fatigue, as initially reported in a Korean patent [106].

### 4.2. Sesquiterpene Lactones (SLs)

#### 4.2.1. Monomeric SLs

**Figure 5:**
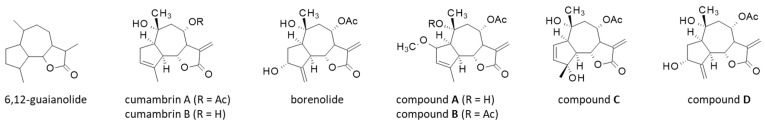
Structures of monomeric sesquiterpene lactones found in *C. boreale*. Borenolide: 8-*O*-acetyl-3,10-dihydroxy-4(15),11(13)-guaiadien-12,6-olide. Compound **A**: 8-acetoxy-2-methoxy-10-hydroxy-3,11(13)-guaiadiene-12,6-olide (R = H). Compound **B**: 8,10-diacetoxy-2-methoxy-3,11(13)-guaiadiene-12,6-olide (R = Ac) [112]. Compound **C**: 8-acetoxy-4,10-dihydroxy-2,11(13)-guaiadiene-12,6-olide [113]. Compound **D**: 8-acetoxy-10-hydroxy-4,11(13)-guaiadiene-12,6-olide [116].

Diverse SLs have been isolated from *C. boreale* extracts, notably guaianolide-type SLs and a few germacranolides. The archetypical SL is cumambrin A present in the plant flowers and frequently used as a phytochemical marker to evaluate the effects of fertilizers on plant growth and metabolite production [22,33,36,39,107]. Both cumambrin A and cumambrin B have been isolated from flowers of *C. boreale* together with the two derivatives angeloylcumambrin B and tigloylcumambrin B (Figure 5). Cumambrin A is largely more abundant than cumambrin B (580 and 75 mg isolated from 2 kg of dried flowers, respectively). Angeloylcumambrin B and tigloylcumambrin B (tested at 100 µg/disc) were found to display antibacterial activities against *P. aeruginosa*, *B. subtilis*, *B. cereus*, *and S. aureus*, (9 mm < diameter of inhibition zone < 12 mm) whereas the parent compound cumambrin B was totally inactive [108,109]. Cumambrins are 6,12-guaianolides found in *Chrysanthemum* species (*C. boreale*, *C. indicum*, *C. zawadskii*, *C. ornatum*) and diverse other plants (e.g., *Eupatorium maculatum*, and *Anthemis carpatica*) [110]. Cumambrin-type SLs are accessible by chemical synthesis via a tandem allylboration/lactonization chemistry [111].

The related compound borenolide and four analogs **A**–**D** have been isolated also from *C. boreale* [112,113,114] (Figure 5). These products have been described but little investigated thus far. Compounds **C** and **D** were found to inhibit nitric oxide release in murine macrophages (IC_50_ = 14 and 7 µg/mL, respectively) [115,116]. These guaiadienolides may well be at the origin of the inhibition of NO production observed with the plant extract [64].

Borenolide is a minor product in the plant compared to cumambrin A. The authors isolated 680 mg of cumambrin A, 8 mg of cumambrin B, and 18 mg of borenolide from 1 kg of dried flowers [113]. Cumambrin A is clearly an abundant product in *C. boreale*. Borenolide has been little studied at present. It is a cytoprotective agent, initially characterized as a apoptosis modulator. It reduced the extent of apoptosis induced in U937 leukemia cells by the antitumor drug etoposide but with a reduced efficacy compared to cumambrins A–B [113]. The pharmacology of cumambrin A has been investigated and two types of bioactivities were evidenced. On the one hand, the product has been found to normalize blood pressure in hypertensive rats upon intravenous administration, in a time-dependent manner. The hypertensive rats gradually recovered normal blood pressure about 4 h after administration of a single dose (10 mg) of the natural product [117,118]. A comparable effect was observed when using isolated rat aortic artery rings precontracted with phenylephrine. The arteries were relaxed to basal tension in the presence of cumambrin A and a synergistic effect occurred when the compound was combined with verapamil. Cumambrin A thus appeared as a potent relaxant of rat aortic smooth muscle, possibly acting as a modulator of Ca mobilization [119].

On the other hand, cumambrin A has been shown to inhibit osteoclast formation and bone resorption in a model of ovariectomized mice, to mimic osteoporosis. The osteoclastogenesis effect was mediated through a suppression of the NF-kB activity mediated by RANKL (receptor activator of NF-kB ligand). Cumambrin A induced a reduction in the number of osteoclasts both in vitro and in vivo [120]. The effect is not specific to cumambrin A. Several sesquiterpene lactones have been shown to inhibit RANKL-induced activation of the NF-κB signaling pathway, notably parthenolide, dehydrocostus lactone, zaluzanin C, micheliolide, cynaropicrin, glaucocalyxin A, and a few other SLs [121,122,123,124,125,126,127]. It is interesting to underline the case of the guaianolide SL cynaropicrin, analogous to cumambrin A, which potently inhibits the transcription activity of NF-κB [128,129]. Its α-methylenebutyrolactone unit is essential to its smooth muscle inhibitory effect [130]. Similarly, dehydrocostus lactone and micheliolide both attenuate osteoclast differentiation and bone resorption by inhibiting NF-κB and other regulators [131,132,133]. This points to a potential class effect associating SL and inhibition of NF-κB activation, but new studies are required to elucidate the molecular mechanism at the origin of the capacity of cumambrin A to limit osteoclastic bone resorption. Mechanisms have been proposed whereby SLs alkylate a key cysteine residue (Cys38) in the p65 subunit of NF-κB (through a Michael-type addition) thereby inhibiting DNA binding [134,135,136]. NF-κB is a driver of proinflammatory gene expression associated with the MAPK pathway. Many proinflammatory genes, such as those coding for COX-2, iNOS, TNFα, IL1β, and others display a binding site for NF-κB in their promoter region. SLs are potent regulators of NF-κB-dependent genes. However, in addition to the NF-κB pathway, SLs regulate other transcription factors (NFAT, STAT3/5, AP1), contributing also to the inflammation process and immuno-regulation [137,138,139].

#### 4.2.2. Dimeric SLs

**Figure 6:**
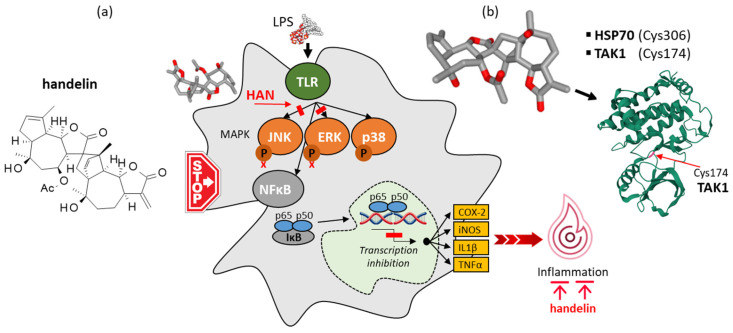
(**a**) Structure and (**b**) mechanism of action of dimeric sesquiterpene lactone handelin found in *C. boreale*. Handelin is also known as yejuhua lactone and chrysanthelide (synonyms). In LPS-activated RAW 264.7 macrophages, handelin (HAN) blocks activation of the NFkB and the MAPK signaling pathway responsible for the induction of pro-inflammatory cytokines. In those cells, HAN (10–40 µM) dose-dependently suppresses the activation (phosphorylation) of JNK and ERK (but not p38) and the upregulation of TNFα and IL1β transcripts [151].

Dimeric, trimeric, and occasionally tetrameric sesquiterpenoids can be found in *Asteraceae* [140,141]. A few oligomeric SLs have been identified in *Chrysanthemum* species, such as the trimeric compound chrysanolide A isolated from flowers of *C. indicum* together with the dimer chrysanolide C and monomer chrysanolide B [142]. The only SL oligomer isolated from *C. boreale* is the guaiane-type sesquiterpenoid dimer called handelin, which is a potent antioxidant and anti-inflammatory agent (Figure 6). The dimeric compound was initially discovered from the plant *Handelia trichophylla*, which also contains the monomer cumambrin A [143,144,145,146]. It was later found in *Chrysanthemum* species, notably *C. indicum* [147], *C. ornatum* [148], and *C. boreale* [149]. The dimeric compound is not cytotoxic but exhibits potent anti-inflammatory effects through the downregulation of mRNA and protein expression of iNOS and COX-2, and the suppression of pro-inflammatory cytokines like TNFα and IL-1β. Its activity has been evidenced in vitro using cultured RAW 264.7 macrophages to show the suppression of JNK and ERK signaling pathways, but also in vivo in a model of carrageenan-induced paw edema in rats. An oral administration of handelin (20 mg/kg) significantly reduced the volume of edema with an efficacy comparable to the reference drug indomethacin [150,151].

**Figure 7:**
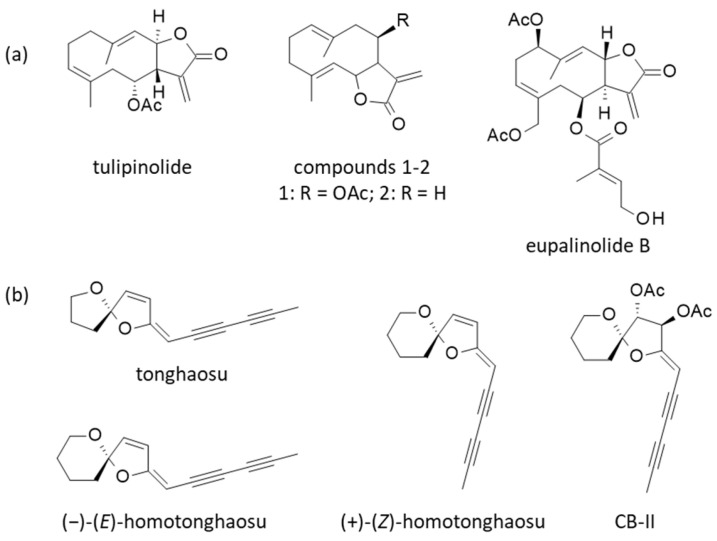
(**a**) Structures of tulipinolide and analogs. (**b**) Structure of the polyacetylenic compound CB-II isolated from *C. boreale* [161,162]. The same compound has been found in *C. lavandulifolium* [164]. CB-II presents a structural analogy with the PPAR-γ agonists (*E*)- and (*Z*)-B-ring-homotonghaosu found in *C. indicum* and *C. morifolium* [171,172].

The anti-inflammatory action of handelin has been well evidenced in a model of elastase-induced emphysema in mice. The capacity of the compound to prevent the development of emphysema was associated with a reduction in NF-κB and AP-1 activation and consequently a downregulation of mediators such as IL-6, iNOS, and MMP-9. Interestingly, the effect was apparently not due to bonding to NF-κB but to the targeting of the ATP-binding pocket of transforming growth factor β-activated kinase 1 (TAK1), which is a kinase upstream of NF-κB [152,153,154]. A very similar effect has been observed with the related anti-inflammatory germacrane SL eupalinolide B, bearing also an α,γ-unsaturated lactone and inactivating TAK1 [155]. This kinase contains a reactive cysteine nucleophile, located near its ATP binding site (Cys174 adjacent to the DFG motif of the kinase activation loop), which can be targeted by electrophilic inhibitors [156,157]. The guaiane-type SL epoxymicheliolide has been shown to bind covalently to Cys174 of TAK1 so as to inhibit NF-κB pro-inflammatory signaling [158]. The same mechanism of action may be invoked with the related SL handelin. Germacranolides structurally related to eupalinolide B have been isolated from flowers of *C. boreale*, such as tulipinolide [159,160], and compounds 1–2 (Figure 7) [109]. An investigation of their mechanism of action as TAK1 inhibitors would be justified.

Handelin displays anti-inflammatory and antioxidant activities. Notably, the compound was shown to prevent UVA- and UVB-induced photoaging of skin fibroblasts through inhibition of reactive oxygen species [161,162]. It not only reduces oxidative damage but also maintains muscle architecture by stabilizing myofilaments and these effects contribute to delaying aging [163]. The compound was shown to reduce skeletal muscle atrophy associated with aging or cachexia [164]. For these reasons, handelin has been proposed as a candidate to treat conditions and pathologies associated with muscle atrophy. A handelin-containing extract of *C. boreale* could be of interest in limiting the extent of muscle wasting in cancers and other pathologies associated with muscle wasting [164]. The idea is interesting but it is worth keeping in mind that, like many SLs bearing a reactive α-methylenebutyrolactone unit, handelin can react with different proteins with exposed cysteine residues. The drug has been shown to react with TAK1 and also with the heat shock protein 70 (Hsp70), which is a multifunctional chaperone essential in maintaining protein homeostasis. Handelin can react with a cysteine residue (Cys306) of Hsp70 so as to activate the chaperone via an allosteric regulation. The effect is beneficial because activation of Hsp70 contributes to modulating neuroinflammation and reduces hypertonic-induced cell death [165,166]. Hsp70 is a key cytoprotective protein and also an important target to reduce the entry of certain viruses into cells, notably for the feline calicivirus (FCV) which replication can be reduced efficiently with handelin (IC_50_ = 2.5 µM) [167]. Hsp70 is involved in diverse pathologies notably in onco-hematology but it is a complex actor with many partners and co-chaperones, considered with a limited druggability. Recently, it was underlined that Hsp70 inhibitors never reached the clinic [168]. All of this is to say that the reactivity of handelin toward proteins like TAK1 and Hsp70 is very interesting but the covalent binding may not be limited to these proteins. Handelin may well react with other proteins bearing exposed cysteines, including metabolism enzymes and diverse signaling molecules. For example, the diSL microlenin bearing the same α-methylene-γ-lactone moiety has been shown to react with inosine monophosphate (IMP) dehydrogenase [169]. The situation is reminiscent of that discussed recently with other series (withanolides) of natural products bearing an α,β-unsaturated carbonyl system capable of reacting with more than 20 proteins [170].

### 4.3. Other Compounds

A few other natural products have been isolated from *C. boreale*, notably the bis(acetylenic) spiroacetal CB-II; however, its stereochemistry has not been clearly identified at present (Figure 7b). It is analogous to the two isomeric bis-acetylenic derivatives (*E*)- and (*Z*)-B-ring-homotonghaosu (1 and 2) found in *C. indicum* and *C. morifolium*, and which display PPAR-γ agonistic effects [171,172]. In another publication (in Korean), a related compound designated CB-L1 (3-(3-methylbutanoly)2-(2,4-hexadivinylidene)-1,6-dioxaspiro [4,5]decane) has been presented as a mild cytotoxic agent, inhibiting the proliferation of five cancer cell lines (IC_50_ = 3.10 µg/mL) [173,174]. Polyacetylenic compounds have been found in other *Chrysanthemum* species [175], notably a diacetoxy derivative found in *C. lavandulifolium* and which seems to be very similar to CB-II (see compound 6 [(−)-(3S,4S,5R)-(E)-3,4-diacetoxy-2-(hexa-2,4-diynyliden)-1,6-dioxa-spiro[4,5]decane] in [176]). In the plant, these polyacetylenic derivatives generally serve as insecticidal agents to protect against plant-feeding predators, notably mites [175,177]. Finally, two additional compounds have been isolated from *C. boreale*: the sterol β-sitosterol and zingerone 4-O-β-D-glucopyranoside [178]. To conclude this phytochemical survey, we can mention a series of biosurfactants isolated from flowers of *C. boreale* but produced by the endophytic fungus *Aureobasidium pullulans*. Three compounds, designated pullusurfactins A-C, have been isolated and their surfactant activity characterized [179]. Other fungi have been found on the plant flowers, notably *Didymella chrysanthemi* responsible for important damages (black blights) [180].

## 5. Discussion

*Chrysanthemum* represent a major plant group owing to its ornamental, social, and economic values. The *Chrysanthemum* Festival (Kikumatsuri in Japanese) is a major autumnal event in Japan to celebrate these plants, their rich history, the symbol they represent, and the large diversity of the species. The *Chrysanthemum* agribusiness is very dynamic in producing countries in Asia, notably in India, Pakistan, Indonesia, Korea, and China [181]. The business relies on cultivated species and farming. New cultivars with novel characteristics (new shape, color, resistance, seasonality) are regularly proposed. One can even find cultivars of edible species such as the flowers of *C. morifolium*, the flowers (Juhua) of which are used as a dietary herbal medicine [182,183]. The flowers of *C. boreale* are also used as an edible natural medicine [22].

In this context, wild *Chrysanthemum* species like *C. boreale* are important because they generally harbor a variety of favorable resistance genes that can be introduced into florist *Chrysanthemums* using molecular breeding technologies [8,9]. *C. boreale* is resistant to one of the most destructive fungal diseases, namely white rust caused by the fungus *Puccinia horiana Henn* [51,184]. Much attention has been paid to wild-type *Chrysanthemum* species and the associated phytochemicals owing to their pharmacological properties, notably their anti-inflammatory activities as mentioned above [185]. *C. boreale* is the archetypal *Chrysanthemum* species of major interest to botanists and pharmacologists. It is a wonderful species that “has been seen everywhere and by everybody” to cite the words of *Madame Chrysanthème* (*Madame Chrysanthème* is a comic opera from André Messager (1893) based on the novel (1888) of the French story writer Pierre Loti (Louis-Marie-Julien Viaud, known as Pierre Loti, 1850–1923), the comic opera from the French compositor André Messager (1893) [186].

Extracts from *C. boreale* can be used for multiple purposes, as depicted in Figure 2 (and detailed in Table 1, Table 2 and Table 3). The pharmacological indications are large, depending on the type of extract. The anti-inflammatory action is probably the most interesting aspect, principally observed with alcoholic extracts. This anti-inflammatory activity has been exploited via a specific patent (WO2011065657 published in 2011) and the product was particularly recommended for the treatment of atopic dermatitis [187]. Alcoholic extracts display essentially anti-inflammatory properties, whereas the essential oil from *C. boreale* was mainly proposed for its anti-oxidant and anti-obesity actions, as mentioned also in a specific patent [188]. However, in other contexts, essential oils from *Chrysanthemum* species can be used also as anti-inflammatory agents [189]. These plant species are particularly rich in volatile terpenoid secondary metabolites, which can be exploited to combat pathologies with an inflammatory component [190]. The flavonoids contained in *C. boreale* extracts represent important active principles. The flavone glycoside linarin deserves special attention due to its marked anti-inflammatory capacity coupled with acetylcholinesterase inhibitory activity. Linarin is exploited as a model compound to design semi-synthetic derivatives active against osteoarthritis and osteoporosis [99].

The major bioactive chemicals found in *C. boreale* are the various monomeric and dimeric sesquiterpene lactones that contribute largely to the anti-inflammatory action of the plant extracts. The case of the dimer handelin is remarkable. The compound could be further exploited as a TAK1 inhibitor to treat pathologies associated with cachexia, such as Duchenne muscular dystrophy [153,191]. Disesquiterpenoids are relatively abundant in nature but handelin represents a unique compound, little explored thus far [192,193]. Recently, handelin and four analogs designated chryindicolides V-W, chrysanolide D, and 8-tigloylchrysanolide D, have been isolated and characterized from the species *C. indicum* [194]. Handelin, but not its analogs, was found to efficiently reduce lipid accumulation and to inhibit ferroptosis induced in AML12 hepatocytes after treatment with palmitic acid and oleic acid (IC_50_ = 6.81 µM compared to 12.21 µM with the reference product simvastatin). The related product chryindicolide O was found to be even more potent (IC_50_ = 4.59 µM) with a marked capacity to bind and to activate the deacetylase Sirtuin 1 (SIRT1) to reduce de novo lipogenesis [194]. This key discovery shed light on these peculiar guaianolide dimers and their interest in treating hepatic steatosis. Altogether, the information reinforces the interest in handelin as a model natural product to design regulators of ferroptosis.

Without a doubt, *C. boreale* has still more beautiful days in front of it, via its contribution to the *Chrysanthemum* Festival and the discovery of bioactive natural products. It is certain that, in our scientific field, the *C. boreale* bio/chemo festival will continue to rally and inspire enough *Chrysanthemum* supporters—botanists, biologists, chemists, and pharmacologists—within the sciences to provide a clear vision of the plant and its medicinal benefits.

## Data Availability

No new data were created.

## Abbreviations

The following abbreviations are used in this manuscript:

ACE	Angiotensin-converting enzyme
AChE	acetylcholinesterase
AE	aqueous extracts
AR	aldose reductase
COX-2	cyclooxygenase-2
EO	essential oil
Hsp70	heat shock protein 70
iNOS	inducible nitric oxide synthase
NF-κB	nuclear factor kappa-light-chain enhancer of activated B cells
NO	nitric oxide
OE	organic extracts
PGE2	prostaglandin E2
ROS	reactive oxygen species
SL	sesquiterpene lactone
SOD	superoxide dismutase
TAK1	transforming growth factor-beta-activated kinase 1
